# A progression analysis of motor features in Parkinson's disease based on the mapper algorithm

**DOI:** 10.3389/fnagi.2023.1047017

**Published:** 2023-02-21

**Authors:** Ling-Yan Ma, Tao Feng, Chengzhang He, Mujing Li, Kang Ren, Junwu Tu

**Affiliations:** ^1^Department of Neurology, Center for Movement Disorders, Beijing Tiantan Hospital, Capital Medical University, Beijing, China; ^2^Department of Neurology, China National Clinical Research Center for Neurological Disease, Beijing, China; ^3^Parkinson's Disease Center, Beijing Institute for Brain Disorders, Beijing, China; ^4^Institute of Mathematical Sciences, ShanghaiTech University, Shanghai, China; ^5^GYENNO Science Co., LTD., Shenzhen, China; ^6^Department of Neurology, HUST-GYENNO Central Neural System Intelligent Digital Medicine Technology Center, Wuhan, China

**Keywords:** progression analysis, Parkinson's disease, mapper algorithm, Markov chain, prediction model

## Abstract

**Background:**

Parkinson's disease (PD) is a neurodegenerative disease with a broad spectrum of motor and non-motor symptoms. The great heterogeneity of clinical symptoms, biomarkers, and neuroimaging and lack of reliable progression markers present a significant challenge in predicting disease progression and prognoses.

**Methods:**

We propose a new approach to disease progression analysis based on the mapper algorithm, a tool from topological data analysis. In this paper, we apply this method to the data from the Parkinson's Progression Markers Initiative (PPMI). We then construct a Markov chain on the mapper output graphs.

**Results:**

The resulting progression model yields a quantitative comparison of patients' disease progression under different usage of medications. We also obtain an algorithm to predict patients' UPDRS III scores.

**Conclusions:**

By using mapper algorithm and routinely gathered clinical assessments, we developed a new dynamic models to predict the following year's motor progression in the early stage of PD. The use of this model can predict motor evaluations at the individual level, assisting clinicians to adjust intervention strategy for each patient and identifying at-risk patients for future disease-modifying therapy clinical trials.

## 1. Introduction

Parkinson's disease is a neurodegenerative disease with a broad spectrum of motor symptoms including bradykinesia, rigidity, resting tremor, and postural and gait impairments (Selikhova et al., 2009). In the clinical course of PD, both linear (Gottipati et al., 2017; Holden et al., 2018) and non-linear progression (Vu et al., 2012; Reinoso et al., 2015) have been reported in the advancement of motor and non-motor symptoms. The substantial heterogeneity in the presentation of clinical phenotypes, genetics, pathology, and disease progression (Foltynie et al., 2002; Selikhova et al., 2009; Ma et al., 2015) and lack of reliable progression markers of neurodegeneration present a major challenge for prediction of progression and accurate prognoses, hampering advances in PD trials, and the clinical routine determining therapeutic efficacy. In an era of increasing focus on individualized management and disease-modifying therapies, there is a need to develop useful tools to predict each patient's motor progression with high accuracy.

The current literature on PD progression consists largely of associative analyses and a few prognostic models. The prognostic models include logistic regression and Bayesian classification models to predict cognitive impairment (Schrag et al., 2017; Hogue et al., 2018; Gramotnev et al., 2019), machine-learning, random survival forests to predict time to initiation of symptomatic treatment (Simuni et al., 2016) and disease progression (Latourelle et al., 2017; Severson et al., 2021). Besides, partial least squares path modeling (PLS-PM), combined with MRI biomarkers, were used to predict progression subtypes and cognitive impairment in prodromal PD (Pyatigorskaya et al., 2021; Rahayel et al., 2021). Based on the Parkinson's Progression Markers Initiative (PPMI) database, we previously built five regression models to predict PD motor progression represented by the coming year's Unified Parkinson's Disease Rating Scale (MDS-UPDRS) Part III score, finding adjusted *R*^2^ values of three different categories of regression model, linear, Bayesian, and ensemble, all reached 0.75 (Ma et al., 2020).

In this study, we propose a new approach to disease progression analysis based on topological data analysis (TDA), or the mapper algorithm to be precise. The mapper algorithm was introduced in Singh et al. (2007) by Singh-Memoli-Carlsson as a way of capturing topological/geometric informations of a point cloud dataset possibly in a high dimensional Euclidean space. Roughly speaking, it may be viewed as an algorithm to compute a given dataset's geometric “shape" by certain combinatorial object which, in the simplest form, may be a graph or a polyhedron. In the case of analyzing patients' data, the method has been successfully implemented in a variety of circumstances (see for example Nicolau et al., 2011; Li et al., 2015; Rossi-deVries et al., 2018; Dagliati et al., 2020).

It is always difficult to predict PD because of great heterogeneity, including subtypes, markers, and various scales. Only by combining clinical presentation and mathematical methods, selecting appropriate parameters and applying appropriate methods can the accuracy of prediction model be improved. Based on PPMI data and our previous predicting models, we aim to improve our multiple dynamic prediction model *via* mapper algorithm in this study. Similarly, general information and classical clinical scales, which are routinely and easily performed in clinical activities, were used to predict motor progression, displayed in the form of the MDS–UPDRS Part III score. These inexpensive and easily readily available clinical data can facilitate widespread implementation of this cost-efficient predictive model in real world applications.

## 2. Materials and methods

### 2.1. Feature selection and data pre-processing

The data were obtained from the PPMI database. The PPMI is an international, multicenter, prospective study designed to discover, and validate biomarkers of disease progression in newly diagnosed PD participants (National Clinical Trials identifier NCT01141023). Each PPMI recruitment site received approval from an institutional review board or ethics committee on human experimentation before study initiation. Written informed consent for research was obtained from all individuals participating in the study. The PPMI database was accessed on December 16, 2022, to obtain data from 943, 379, 324, 256, 268 visits for five consecutive years. For up-to-date information on the study, please visit www.ppmi-info.org.

Since the mapper algorithm is a way of computing the “shape" of a given data set in ℝ^*N*^, if the dimension *N* is too large while the data set is relatively small, the shape would only be a collection of sparse points. Thus, our first step uses a topological method to reduce the number of features introduced in Kraft (2016). The idea behind this feature selection method is that we could eliminate a feature if it does not cause a big change in the underlying topology (calculated using persistent homology) of the data sets. We refer to the article (Kraft, 2016) for more details.

In our case, for the feature selection, we first consider the following listed 29 features mostly used in Ma et al. (2020). We have added a feature “symptom” which is given by the sum symptom1, symptom2, symptom3, and symptom4, with

- Symptom1: Initial symptom (at diagnosis)—Resting Tremor
- Symptom2: Initial symptom (at diagnosis)—Rigidity
- Symptom3: Initial symptom (at diagnosis)—Bradykinesia
- Symptom4: Initial symptom (at diagnosis)—Postural Instability

All these variables are binary such that it is 0 if No symptom or unknown; 1 if Symptom present at diagnosis.

In general, the features we consider are inexpensive and easily readily available clinical data. Each of the coordinates is normalized to [0, 1]. In the coordinate given by the UPDRS III score, we also performed a clamping at 0.7.^1^ These features are listed as follows:

*updrs*3*, age, NP*1*APAT, scopa, YEAR, NP*1*FATG, moca, symptom, NP*1*ANXS, gds, PD_MED_USE, symptom*2*, NP*1*HALL, ageonset, NP*1*COG, NP*1*DPRS, rem, ess, symptom*1*, DOMSIDE, “PATNO,” gen, symptom*4*, fampd_new, NP*1*DDS, duration, td_ pig, quip, symptom*3.

In the above we have ordered the features according to their Pearson's correlation coefficients with the UPDRS III score. An important point to note is that, excluding the UPDRS III score itself, the maximal of these Pearson's correlation coefficients is 0.27, which shows that their correlation with the UPDRS III score is in general highly non-linear. This is an ideal context to use our topological data analysis (TDA) method as it is a tool developped to handle non-linear correlations.

**Figure 1:**
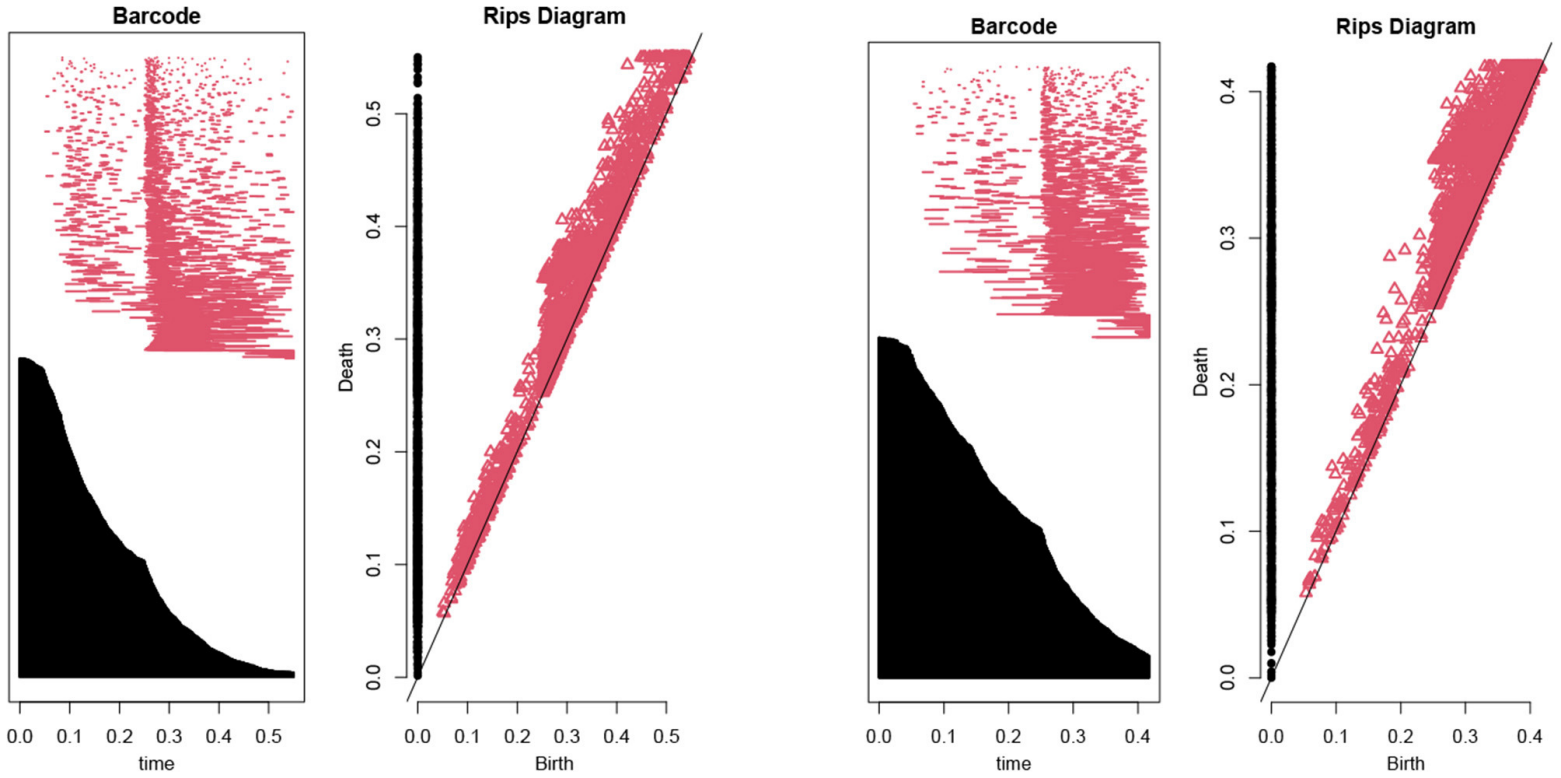
The barcodes and rips diagrams both illustrate the persistent homology (Lum et al., 2013) of a given dataset. The two pictures compare the persistent homology in the case of seven features with the case of eight features. Observe that the right hand side has considerably more persistence (in both black and red markings) compared with the left hand side.

Then, we use the persistent homology to reduce the number of features (Kraft, 2016). In our case, Figure 1 illustrates the persistent homology, when passing from the first eight features to seven features, has a big difference. This tells us we should stop eliminating features. The remaining 8 selected features are listed as in Table 1.

**Table 1:** List of selected features.

**Features**	**Meaning**
Age	Age of patient when data is collected
updrs3	UPDRS III score (OFF)
np1apat	APATHY
np1fatg	FATIGUE
np1anxs	ANXIOUS MOOD
Moca	Montreal cognitive assessment (MOCA) score
Scopa	Scales for outcomes in Parkinson's disease (SCOPA)-AUT total score
Symptom	Symptom1 + Symptom2 + Symptom3 + Symptom4

From the PPMI data, we select these features for each patient's data to form a point cloud *S*^PPMI^ ⊂ ℝ^8^, of size |*S*^PPMI^| = 2, 389, consisting of 481 distinguished patients.

### 2.2. The mapper algorithm

The mapper algorithm introduced by Singh-Memoli-Carlsson (Singh et al., 2007) is a method to analyze high dimensional data based on ideas from topology—a branch of mathematics to study complex shapes of geometric objects.

**Figure 2:**
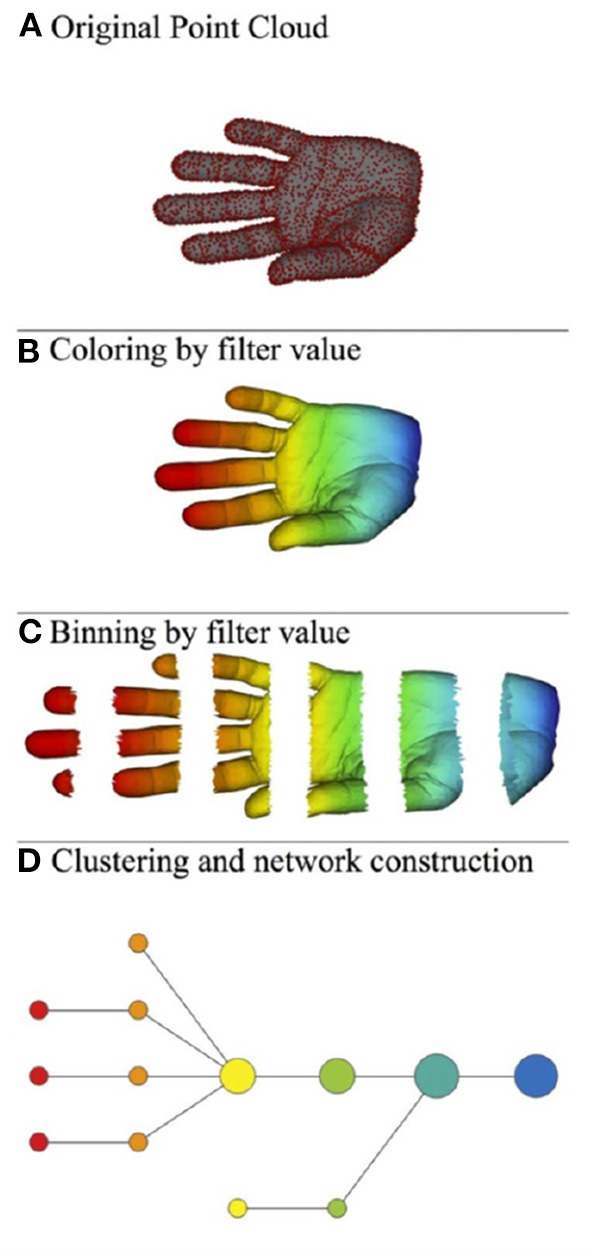
Illustration of the mapper algorithm in the case of a point cloud *S* in ℝ^2^, and with the filter function given by the horizontal projection. The outcome of this algorithm is the bottom graph. **(A)** Original point cloud, **(B)** Coloring by filter value, **(C)** Binning by filter value, and **(D)** Clustering and network construction.

Roughly speaking, the mapper algorithm consists of several steps as illustrated in Figure 2.

(A) A point cloud data *S* ⊂ ℝ^*N*^ whose topological/geometric properties we would like to study.
(B) A choice of *d* filter functions on *S*.
  $$f=\left(f_{1},\ldots ,f_{d}\right):S\to {\mathbb{R}}^{d}.$$
(C) Choose a covering of the image of *f* by boxes:
  $$\text{Im}\left(f\right)\subset \underset{\alpha }{⋃}B_{\alpha }$$
  with each *B*_α_ a box in ℝ^*d*^. Put the data *S* into overlapping bins by taking $S_{\alpha }:=f^{-1}\left(B_{\alpha }\right)$.
(D) Cluster each bin *S*_α_ and create a simplicial complex recording the intersection pattern between the clusters. Often a truncated version is used as mapper's output: the result yields a graph whose vertices correspond to the clusters, and an edge is created whenever two clusters have non-empty intersection.

### 2.3. Construction of Markov chains

We shall apply the mapper algorithm to the point cloud *S*^PPMI^ from the previous subsection. Recall that *S*^PPMI^ ⊂ ℝ^8^ is the sample space of patients' data extracted from the raw PPMI data. Using the mapper algorithm, assume that we have obtained *m* clusters *C*_1_, …, *C*_*m*_ so that $S^{\text{PPMI}}=C_{1}\cup \cdots \cup C_{m}$. Note that these clusters can possibly intersect with each other.

Let *P* ⊂ *S*^PPMI^ × *S*^PPMI^ be a subset. We proceed to use *P* to obtain a Markov chain on the set of clusters *C*_1_, …, *C*_*m*_. For a pair of data (*x, y*) ∈ *P*, if *x* ∈ *C*_*i*_ and *y* ∈ *C*_*j*_, we consider it as an arrow from the cluster *C*_*i*_ to *C*_*j*_. This yields a multi-graph (possibly with multiple edges between vertices) whose vertices are the clusters *C*_1_, …, *C*_*m*_. Then we use informations of this multi-graph to obtain a Markov matrix. More precisely, for each pair of indices (*i, j*) with 1 ≤ *i, j* ≤ *m*, we define


(1) $$\;M_{ij}^{P}:\;=\left\{ \begin{array}{l} \frac{\left|\{(x,y)\in P|x\in C_{i},y\in C_{j}\}\right|}{\left|\{(x,y)\in P|x\in C_{i}\}\right|}\text{ if }\{(x,y)\in P|x\in C_{i}\}\neq \text{∅}\\ \delta_{ij}\text{ if }\{(x,y)\in P|x\in C_{i}\}=\text{∅} \end{array}\right.$$

#### 2.3.1. Computing expected growth

For each 1 ≤ *j* ≤ *m*, denote by

$$E_{j}:=\frac{1}{\left|C_{j}\right|}\cdot \underset{y\in C_{j}}{\sum }\text{updrs}3\left(y\right)$$

the expected value of the UPDRS III score of the cluster *C*_*j*_.

The expected growth of a patient's UPDRS III of a fixed PD medication type *i* is computed as follows.

(1) Fix the medication type index 0 ≤ *i* ≤ 7. Consider the distribution of patients with medication type *i* in each cluster, i.e., for each 1 ≤ *j* ≤ *m* denote by
  $$\begin{array}{r} d_{j}:=|\{x\in C_{j}|\text{The data x is from a patient with PD}\\ \left\{\text{medication typei}.\}\right| \end{array}$$
(2) Form the initial probability vector that a type *i* patient belongs to each cluster:
  $$w:=\frac{1}{\sum_{j=1}^{m}d_{j}}\left(d_{1},\ldots ,d_{m}\right).$$
(3) The expected growth in 1 year of such a patient is then computed by
  $$\Delta :=\overset{m}{\underset{j=1}{\sum }}w_{j}\cdot \Delta_{j}$$
  where ${\text{Δ}}_{j}=\overset{m}{\underset{l=1}{\sum }}M_{jl}^{P_{i}}\left(E_{l}-E_{j}\right)$ is the expected growth of the UPDRS III score for a patient in the cluster *C*_*j*_.

### 2.4. Prediction models

As a second application, we use the Markov chains obtained in the previous paragraph to build a prediction model for a patient's UPDRS III score in the next year. This is done in several steps:

(a) Given a patient's current year data *x* ∈ ℝ^8^, we first produce an initial probability vector
  $$v=\left(v_{1},v_{2},\ldots ,v_{m}\right)$$
  where recall that *m* is the number of clusters in the mapper output. See Equation (2) for the definition of *v*. In other words, *v*_*j*_ is the probability of *x* lie inside the *j*-th cluster *C*_*j*_.
(b) Then compute the action of the Markov chain on the vector *v* to obtain
  $$v^{†}:=v\cdot M^{P}=\left(v_{1}^{†},\ldots ,v_{m}^{†}\right)$$
(c) The predicted UPDRS III score is then equal to
  $$p\left(x\right):=\overset{m}{\underset{j=1}{\sum }}v_{j}^{†}\cdot E_{j}$$
  where as before $E_{j}:=\frac{1}{\left|C_{j}\right|}\cdot \underset{y\in C_{j}}{\sum }updrs3\left(y\right)$ is the expected value of the UPDRS III score of the cluster *C*_*j*_.

The first step (*a*) needs more explanation, and is realized as follows. Fix a positive integer *μ* > 0, and a positive real number *σ* > 0. We find the first *μ* nearest point $a_{1},\ldots {\;a}_{\mu }\in S^{\text{PPMI}}$ to the given point *x*. Then use the equation


$$c\cdot \overset{\mu }{\underset{k=1}{\sum }}e^{-\frac{||x-a_{k}{||}^{2}}{\sigma^{2}}}=1$$

to determine a constant *c*. For each 1 ≤ *k* ≤ *μ*, the point *a*_*k*_ may belong to several clusters. Denote its multiplicity by


$$l_{k}:=|\left\{1\leq i\leq m|a_{k}\in C_{i}\right\}|$$

At this point, it is tempted to set the initial probability vector by formula


$$v_{i}=\underset{1\leq k\leq \mu ,a_{k}\in C_{i}}{\sum }\frac{1}{l_{k}}\cdot c\cdot e^{-\frac{||x-a_{k}{||}^{2}}{\sigma^{2}}}.$$

However, observe that already in the definition of *M*^*P*^ (see Equation 1), it is possible that a cluster *C*_*i*_ is not the source of any arrows, i.e.,


$$\left\{\left(x,y\right)\in P|x\in C_{i}\right\}=\emptyset .$$

In this case, it is not possible to use such type of clusters to make predictions for the next year's data. Thus, we set the initial probability at such a cluster by zero, and rescale the resulting vector by a constant to obtain the desired initial probability vector. Explicitly, we set the initial probability vector *v* = (*v*_1_, *v*_2_, …, *v*_*m*_) by


(2) $$v_{i}:\;=\left\{ \begin{array}{l} \text{const}\cdot \sum_{1\leq k\leq \mu ,\;a_{k}\in C_{i}}\frac{1}{l_{k}}\cdot c\cdot e^{-\frac{||x-a_{k}{||}^{2}}{\sigma^{2}}}\text{ if }\{(x,y)\in P|x\in C_{i}\}\neq \text{∅}\\ 0\text{ if }\{(x,y)\in P|x\in C_{i}\}=\text{∅} \end{array}\right.$$

In this paper, we shall fix the parameters to be *μ* = 14 and *σ* = 0.0378.

## 3. Results

### 3.1. Mapper outputs

**Figure 3:**
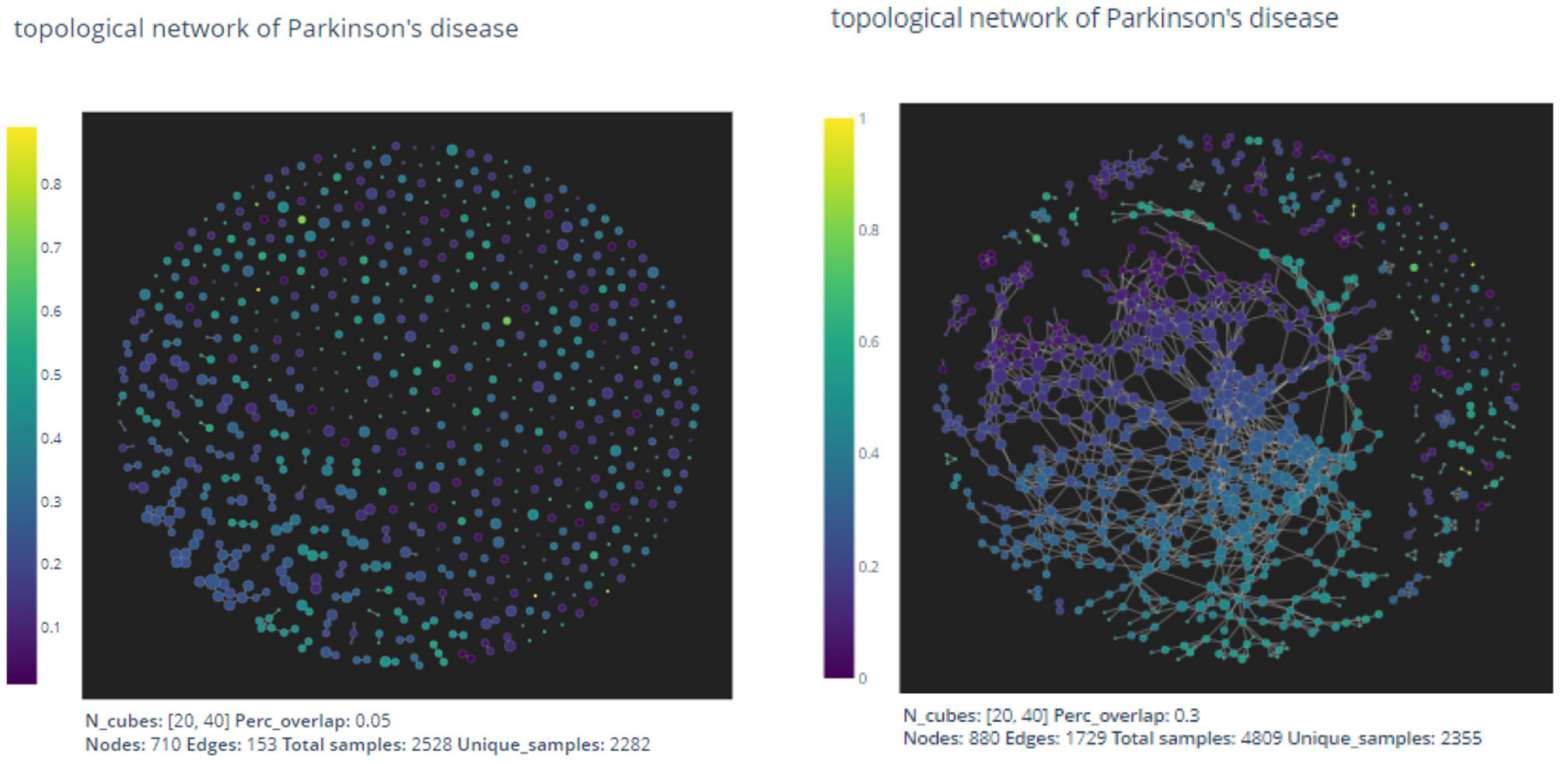
The pictures are out-puts of the mapper algorithm with parameters given by *n*_1_ = 20, *n*_2_ = 40, *p* = 0.05 and *n*_1_ = 20, *n*_2_ = 40, *p* = 0.3, respectively.

We apply the Kepler mapper program 1.4.1 (van Veen et al., 2019a,b) to the point cloud set *S*^PPMI^ with a 2-dimensional filter function


$$f=\left(\text{age},\text{updrs}3\right):S\to {\mathbb{R}}^{2}$$

given by two coordinate projections in the direction of “age" and “updrs3." The output graph is shown in Figure 3.

As expected by the formation of the mapper algorithm, larger percentage of overlaps naturally leads to more non-empty intersections between clusters, and hence the graph on the right appears to have more edges than the left one.

In the two dimensional mapper algorithm, there are three parameters to choose:

- *n*_1_: Number of intervals in the “updrs3" direction.
- *n*_2_: Number of intervals in the “age" direction.
- *p*: Percentage of overlaps in both direction.

There exists no general method to determine appropriate parameters in the mapper algorithm. In the next section, we shall use the mapper output to construct a prediction model for the UPDRS III scores of patients. We then use the precision value of the resulting prediction model to evaluate and thus optimize the parameters.

### 3.2. Markov chains

From the PPMI data, there are eight different types of patients according to their usage of PD medications, as shown in Table 2.

**Table 2:** List of medication types.

**Type index**	**Medication**
0	Unmedicated
1	Levodopa
2	Dopamine agonist
3	Other
4	Levodopa + other
5	Levodopa + dopamine agonist
6	Dopamine agonist + other
7	Levodopa + dopamine agonist + other

**Figure 4:**
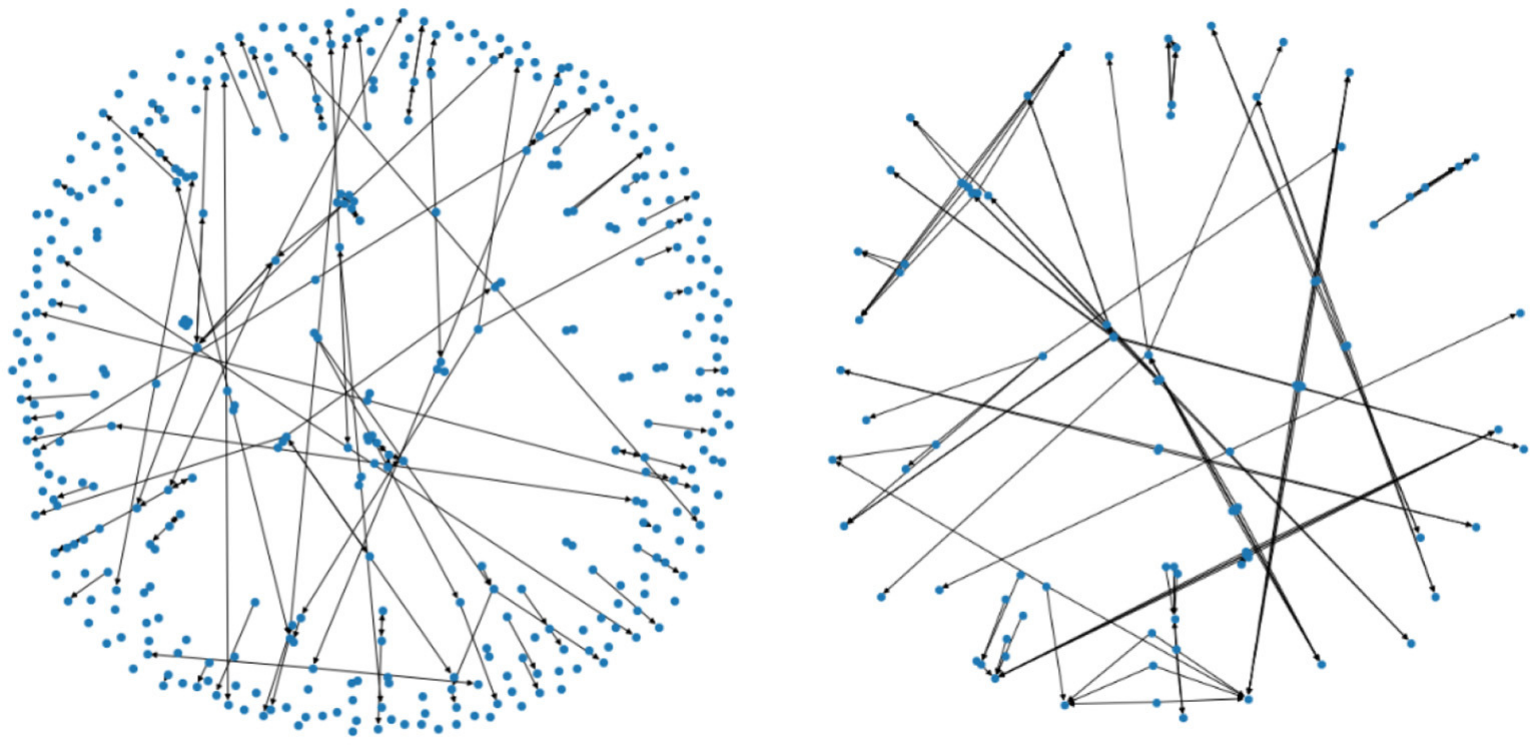
The two figures illustrate two Markov chains associated with medication type 0 and 1, respectively. Its nodes are derived from the outputs of the mapper algorithm.

Denote by $P_{i}\subset S^{\text{PPMI}}\times S^{\text{PPMI}},0\leq i\leq 7$ the subset consisting of pairings (*x, y*) such that the data *x* and *y* are two consecutive years' data from the same patient (i.e., a progression by 1 year), and that the patient's usage of PD medication is of type *i* in the above table. For *i* = 0 and *i* = 1 we have depicted the corresponding two Markov chains in Figure 4 (with mapper parameters set to be *n*_1_ = 20, *n*_2_ = 40, *p* = 0.05).

### 3.3. PD medication type analysis

As a first application of the Markov chains $M^{P_{i}}$ obtained from the previous paragraph. We use it to compute the expected growth of a patient's UPDRS III score according to the patient's PD medication type. The computed results are shown in Table 3.

**Table 3:** Expected growth of UPDRS III score associated with different medication types.

**PD medication type index**	**Expected growth of UPDRS III score**
0	2.17
1	2.24
2	2.51
3	3.37
4	2.19
5	0.30
6	0.88
7	1.85

The expected growth of PD patients with a particular type of medication certainly may depend on the particular choice of medication to begin with. Thus, it makes sense to perform an un-biased comparison with what happens if the medication type *i*≠0 group of patients were not given any medication. To do this, consider the following probability distribution (*p*_1_, …, *p*_*m*_) on the set of clusters defined by


$$p_{j}:=\frac{\text{type }i\;\text{patients in }C_{j}}{\text{all type }i\;\text{patients}}.$$

We can calculate the expected growth viewed as un-medicated patients under same distribution using the Markov chain $M_{jk}^{P_{0}}$:


$$\Delta_{i}^{\prime }:=\underset{j}{\sum }\underset{k}{\sum }p_{j}M_{jk}^{P_{0}}\left(E_{k}-E_{j}\right)$$

The difference between ${\text{Δ}}_{i}^{\prime }$ and the actual expected growth Δ_*i*_ would measure the benefit of the *i*-th type medication to reduce the growth of patients' UPDRS III scores. Calculations demonstrate solid medication effects in the cases of type 4, 5, and 6, as shown in Table 4. Observe that patients in medication type 5 and 6 have relatively small expected growth of UPDRS score in Table 3. The un-biased analysis gives at least a partial explanation for this: for these two groups of patients medication effects are rather significant.

**Table 4:** Un-biased medication effects in medication type 4, 5, and 6.

**Medication type *i***	**Δ_*i*_**	** ${\text{Δ}}_{i}^{\prime }$ **	** ${\text{Δ}}_{i}^{\prime }-{\text{Δ}}_{i}$ **
4	2.19	2.50	0.31
5	0.30	1.30	1
6	0.88	2.29	1.41

### 3.4. Statistics of the prediction models

To test the validity of our prediction model described above, for each PD medication type index 0 ≤ *i* ≤ 7, we perform a statistical study of its accuracy as follows.

(1) First take out a point $\left(x_{0},y_{0}\right)\in P_{i}\subset S^{\text{PPMI}}\times S^{\text{PPMI}}$, run the prediction model with *S*−{*x*_0_} to obtain the predicted next year's UPDRS III score *p*(*x*_0_).
(2) Do step (1) for all points (*x*_0_, *y*_0_) in *P*_*i*_. Then perform a statistical study between the predicted score *p*(*x*_0_) with the actual next year's score *y*_0_.

Table 5 shows the statistics of our prediction models in each PD medication type. The *R*^2^ score, MAE, MSE and Max Error are well-known statistical measures. We explain the last column “hit percentage." In the evaluation of UPDRS III score (a total of 132 points), medical experiences usually permits a variation of ±5 points. In our data set *S*^PPMI^, the difference between maximal score and the minimal score is 80. Since we have normalized this score to [0, 1], a variation of ±5 absolute points would corresponds to ±0.0625 after normalization. The “hit-in percentage” is the percentage of the prediction score *p*(*x*_0_) “hit-in” the interval [*y*_0_−0.0625, *y*_0_+0.0625] since we regard such a prediction as being a successful one.

**Table 5:** Statistics of the TDA method.

**Medication**	***R*^2^ score**	**MAE**	**MSE**	**Max error**	**Hit in percentage %**
0	0.67	–0.0121	0.00597	0.216	62.6
1	0.726	–0.00807	0.00607	0.222	82.7
2	0.966	–0.00542	0.000396	0.0127	97.0
3	0.872	–0.000186	0.0015	0.14	88.4
4	0.642	–0.0142	0.00691	0.247	77.6
5	0.749	–0.012	0.00515	0.217	87.7
6	0.499	–0.0023	0.00698	0.274	78.6
7	0.953	0.000792	0.00073	0.0663	94.3

### 3.5. Comparison with classical regression methods

The statistics shown above should be compared with an earlier prediction model (Ma et al., 2020). In *loc. cit.* the authors used classical methods such as Linear Regression, Bayesian Regression, and so on. For example, in the case of *P*_1_, the comparison of statistics of our TDA method with classical methods is shown in Table 6.

**Table 6:** Comparison between statistics of the TDA method with classical regression methods.

	***R*^2^ score**	**MAE**	**MSE**	**Max error**	**Hit in percentage %**
TDA	0.726	–0.00807	0.00607	0.222	82.7
Linear regression	0.607	0.0632	0.00704	0.384	55.7
Ridge regression	0.642	0.0693	0.00794	0.315	46.8
Bayesian regression	0.689	0.0635	0.0069	0.283	55.5
Random forest	0.733	0.0372	0.00593	0.562	78.6
Gradient boosting	0.783	0.0364	0.00481	0.311	79.2

This shows that the mapper algorithm combined with Markov chain construction is more efficient than the more classical regression methods in the study of progression analysis of Parkinson's disease.

## 4. Discussion

In this study, we develop a new predictive model for motor progression in patients with early PD by mapper algorithm, which we report 62.5% accuracy in the group of un-medicated patients (Medication type 0); while in other medication types, the accuracy increased, fluctuating between 77.6 and 97% (Medication type 1–7). Also, we compared different methods in the analysis of PD progression and found that mapper algorithm combined with Markov chain construction is more efficient than the more classical regression methods. This prediction model is an upgrade of our previous prediction model, which improves the accuracy and has better stability. Our findings indicate that the models can practically predict the MDS-UPDRS Part III score of the coming year based on the clinically available characteristics obtained in the current year.

There are a growing number of clinical prediction models of the progression of PD, which vary from the choices of predictive values according to different objectives. Latourelle et al. developed and validated a comprehensive multivariable prognostic model based on the PPMI database (Latourelle et al., 2017). In this model, they obtained a *R*^2^ of 41% in PPMI database and 9% in LABS-PD database that used for external validation. This reduction of *R*^2^ could be offset by increasing the sample size. As in Lu et al. they developed a progression model based on the videos of MDS-UPDRS tests to estimate the motor severity of PD, in which they obtained a classification accuracy of 72% and F1-score of 0.51 (Lu et al., 2021).

Eight variables were enrolled in this model, including age, MDS-UPDRS III, NP1 apathy score, NP1 fatigue score, NP1 anxiety score, MOCA, SCOPA-AUT, and initial symptoms. These variables contain quantification of motor (MDS-UPDRS III) and non-motor symptoms (apathy, cognitive dysfunction, fatigue and anxiety), all of which contribute to the progression of PD.

Previous studies have identified that cognitive impairment at baseline is correlated with faster disease progression and greater motor impairment (Velseboer et al., 2013; Fereshtehnejad et al., 2015; Reinoso et al., 2015). Apart from UPDRS values, signs of cognitive decline, orthostatic hypotension and rapid eye movement sleep behavior disorder at baseline, could also suggest a much faster decline in motor symptoms. An increase in L-dopa non-responsive symptoms, which suggest a diffuse destruction of extra-nigrostriatal pathways in parallel with the nigrostriatal pathway (Velseboer et al., 2013) may in part explain the situation.

Overall, PD is a neurodegeneration disease and all the patients suffer from progressive aggravation. The expected growth of motor score varies greatly due to different medication types. The rate of progress of patients with no medication is 2.17 per year, which is representative of PD's natural course. Anti-PD drugs can improve patients' motor symptoms, while the expected growth of UPDRS III score in patients taking medicine is lower than type 1. We also found the expected growth of UPDRS III score in groups 5 (levodopa + dopamine agonist) and 6 (dopamine agonist + other) is lower than other types, indicating that dopamine agonists might improve motor dysfunction better or exist potential disease-modifying effect. However, given the complexity of drugs regulation and interactions with patients, further interpretation should be given cautiously. In addition, according to the type of medication used by the patients, the accuracy of prediction model in the patients taking the anti-PD medication was improved compared to patients with no medication, ranging from 77.6 to 97%. The reason is that in the type 0 case, patients' UPDRS III score could experience a “jumping" phenomenon, thus making our continuous topological method not as effective as in the case of other medication types. In fact, identifying features of this jumping phenomenon is itself an interesting question which we plan to further investigate in a future work.

There are also some limitations in this study. First, the variability and subjectiveness of measures of the motor and non-motor scores within the PPMI dataset may exist. Second, due to limited PD patients, only uniform predictions across subtypes were made without consideration of PD subtypes. Third, we just predict the MDS-UPDRS Part III total score in the predict model, and no subdivision prediction was made for a single item or symptom category score (such as limb rigidity, central axis slowing, tremor, gait, etc.). Finally, our analysis was based on the early stage of PD. As a result, this model cannot be apply to patients with advanced PD for motor prediction.

In this study, by using mapper algorithm, we apply relatively fewer parameters to achieve better results than the previous models, provide accuracy in the range of 62.5 − 97.0% in predicting motor progression depending on different medication types. The use of this model can predict motor evaluations at the individual level, assisting clinicians to adjust intervention strategy for each patient and identifying at-risk patients for future disease-modifying therapy clinical trials.

## Data availability statement

Publicly available datasets were analyzed in this study. The datasets presented in this study can be found in online repositories. The names of the repository/repositories and accession number(s) can be found at: www.ppmi-info.org.

## Author contributions

L-YM: research directions throughout the process and provides medical advise for feature selection together with TF. TF: medical advise for feature selection, suggest to produce practical applications of the algorithm, and such as comparing different medication types using the algorithm. CH: algorithm implementations and coding. ML: data pre-processing. KR: current collaboration and discussions throughout different stages of the program. JT: algorithm implementations involved in the program. All authors contributed to the article and approved the submitted version.

## Footnotes

^1^The reason for clamping is that very rarely we have patients with UPDRS III score > 0.7 after normalization.
