# Corrigendum: A progression analysis of motor features in Parkinson's disease based on the mapper algorithm

**DOI:** 10.3389/fnagi.2023.1174022

**Published:** 2023-03-28

**Authors:** Ling-Yan Ma, Tao Feng, Chengzhang He, Mujing Li, Kang Ren, Junwu Tu

**Affiliations:** ^1^Department of Neurology, Center for Movement Disorders, Beijing Tiantan Hospital, Capital Medical University, Beijing, China; ^2^Department of Neurology, China National Clinical Research Center for Neurological Disease, Beijing, China; ^3^Parkinson's Disease Center, Beijing Institute for Brain Disorders, Beijing, China; ^4^Institute of Mathematical Sciences, ShanghaiTech University, Shanghai, China; ^5^GYENNO Science Co., LTD., Shenzhen, China; ^6^Department of Neurology, HUST-GYENNO Central Neural System Intelligent Digital Medicine Technology Center, Wuhan, China

**Keywords:** progression analysis, Parkinson's disease, mapper algorithm, Markov chain, prediction model

In the published article, there was an error. We made a mistake in describe variables in the predictive model.

A correction has been made to the **Discussion**, Paragraph 3. This sentence previously stated:

“Eight variables were enrolled in this model, including age, MDS-UPDRS III, NP1 apathy score, NP1 fatigue score, NP1 anxiety score, MMSE, and initial symptoms.”

The corrected sentence appears below:

“Eight variables were enrolled in this model, including age, MDS-UPDRS III, NP1 apathy score, NP1 fatigue score, NP1 anxiety score, MOCA, SCOPA-AUT, and initial symptoms.”

In the published article, there was an error. The description of the rate of progress in patients with no medication is ambiguous.

A correction has been made to the **Discussion**, Paragraph 5. This sentence previously stated:

“The rate of progress of patients with no medication is faster, which is representative of PD's natural course.”

The corrected sentence appears below:

“The rate of progress of patients with no medication is 2.17 per year, which is representative of PD's natural course.”

The authors apologize for this error and state that this does not change the scientific conclusions of the article in any way. The original article has been updated.

